# Zeolites as Carriers of Antitumor Ribonuclease Binase

**DOI:** 10.3389/fphar.2019.00442

**Published:** 2019-05-03

**Authors:** Vera Khojaewa, Oleg Lopatin, Pavel Zelenikhin, Olga Ilinskaya

**Affiliations:** ^1^Department of Microbiology, Institute of Fundamental Medicine and Biology, Kazan Federal University, Kazan, Russia; ^2^Department of Mineralogy and Lithology, Institute of Geology and Petroleum Technologies, Kazan Federal University, Kazan, Russia

**Keywords:** zeolites, chabazite, clinoptilolite, natrolite, cytotoxic RNAse, binase, immobilization

## Abstract

Natural and synthetic zeolites have many applications in biomedicine and nutrition. Due to its properties, zeolites can absorb therapeutically active proteins and release them under physiological conditions. In this study we tested the clinoptilolite, chabazite, and natrolite ability to be loaded by antitumor ribonuclease binase and the cytotoxicity of the obtained complexes. We found the optimal conditions for binase loading into zeolites and established the dynamic of its release. Cytotoxic effects of zeolite-binase complexes toward colorectal cancer Caco2 cells were characterized after 24 and 48 h of incubation with cells using MTT-test. Zeolites were toxic by itselfs and reduced cells viability by 30% (clinoptilolite), 40% (chabazite), and 70% (natrolite) after 48 h of incubation. Binase complexes with clinoptilolite as well as chabazite always demonstrated enhanced toxicity (up to 57 and 60% for clinoptilolite and chabazite, respectively) in comparison with binase and zeolites separately. Our results contribute to the perspective development of binase-based complexes for therapy of colorectal cancer for or the treatment of malignant skin neoplasms where the complexes can be used in pasty form.

## Introduction

There are about 40 naturally occurring tectosilicate minerals in zeolite group, the most commonly mined isometric forms include chabazite and clinoptilolite, the fibrous form is mainly represented by natrolite. Chemical differentiation of zeolites is related to the ratio of SiO_2_/Al_2_O_3_ and water content. Zeolites with an Al/Si ratio of 0.20–0.40 are leafy, others with an Al/Si ratio up to 0.50 are isometric or mostly isometric, and with an Al/Si ratio of 0.60–1.00 are predominantly fibrous. The crystalline structure of zeolites is formed by tetrahedral SiO_2/4_ and AlO_2/4_ groups, united into a three-dimensional framework pierced by cavities and channels which size is 0.2–1.5 nm. The internal cavities and the channels are filled with molecules of water. The open frame-cavity structure of zeolites has a negative charge, which is compensated by counterions (metal, ammonium, alkylammonium, and other cations).

Zeolites are capable to exchange cations and reversible dehydrate. Pores in zeolite let small molecules pass through but trap larger ones; that is why they are referred as molecular sieves. Alumina-rich zeolites are attracted to polar molecules such as water, while silica-rich zeolites work better with nonpolar molecules. Advances in material synthesis lead to engineering of hierarchically organized zeolites with multilevel pore architecture which combine unique chemical functionality with efficient molecular transport (Mitchell et al., 2015). Natural and synthetic zeolites are used as drying agents, as detergents, and in water and air purifiers. Zeolites are also marketed as dietary supplements to treat cancer, diarrhea, autism, herpes, and hangover, and to balance pH and remove heavy metals in the body. *In vivo* studies, micronized zeolite has been shown to reduce the spread of cancer and increase the effect of the chemotherapy drug doxorubicin (Zarkovic et al., 2003). Up today, zeolites have not been studied as an anticancer drug in human clinical trials. A review by Memorial Sloan-Kettering Cancer Center concluded that none of the benefits seen in animals occurs in humans^1^. However, different zeolite forms must be distinguished: fibrous mordenite is not allowed for medical use, erionite inhalation toxicity is associated with high incidence of malignant mesothelioma (Elmore, 2003; de Assis et al., 2014). At the same time, TMAZ^®^, a natural isometric zeolite clinoptilolite with enhanced physicochemical properties, is the basis of the dietary supplements Megamin^®^ and Lycopenomin^®^ (“Tribo Ming,” Croatia), which have demonstrated antioxidant activity in humans. Litovit^®^ (“Nov,” Novosibirsk, Russia) that removes heavy metals and has radioprotective properties is also manufactured on the basis of clinoptilolite. The composition synthesized from naturally occurring non-toxic zeolites was patented in United States against buccal mucosa and lung squamous epithelial cell cancers (Kaufman, 2001).

Taken together, this data indicates that adsorptive and ion-exchange properties of some zeolites could be applied in medical practice. In our study, several zeolites allocated as possible candidates for loading of anticancer therapeutics. We tested isometric clinoptilolite and chabazite ability to absorb therapeutic protein and realize it, in comparison to this ability of fibrous natrolite. We chose binase (ribonuclease (RNase) from *Bacillus pumilus*) as a therapeutic protein. RNases are potential antitumor drugs due to their cytotoxicity and due to their influence at some tumor cells functions. RNases have demonstrated the ability to overcome multidrug resistance and to enhance the cytotoxicity of a variety of anticancer agents (Suri et al., 2007; Zelenikhin P.V. et al., 2016). Binase triggers apoptotic response in cancer cells expressing RAS oncogene which is mutated in a large percentage of prevalent and deadly malignancies (Ilinskaya et al., 2001; Cabrera-Fuentes et al., 2012, 2013). Other microbial RNases, cationic mutants of RNAse Sa, for example, possess similar selective activity to oncotransformed cells (Ilinskaya et al., 2002). The specific antitumor effect of binase toward RAS-transformed cells is due to its direct binding of RAS protein and inhibition of downstream signaling (Ilinskaya et al., 2016). The expression of oncogenes, in particular, AML1-ETO and *kit*, was shown to determine the selective sensitivity of cells to the binase action. Moreover, the anti-metastatic effect of binase was demonstrated in animal models. Binase at doses of 0.1–1 mg/kg, which produced effective suppression of tumor growth and metastasis, showed positive effect on the liver of tumor-bearing mice expressed in a significant reduction of the liver parenchyma destructive changes, and return to the normal level the liver regenerative potential (Sen’kova et al., 2014). Thus, this bacterial RNase can be considered a perspective antitumor agent because of its targeted activity toward certain oncogenes expressing cancer cells.

The present study is aimed at the search for a biocompatible mineral carrier that allows the safe delivery and long-term action of binase needed for treatment of *ras*-expressing malignances, especially colorectal cancer. The delivery of proteins to the intestine is known to be complicated by their degradation in digestive tract with subsequent loss of therapeutic activity. Therefore, the prolonged release of antitumor agents from composite pills or rectal suppositories can provide certain advantages. Similarly, these advantages are inherent in therapeutic application of pasty form for the treatment of malignant skin neoplasms. Here, we found the optimal conditions for binase loading into zeolites and established the dynamic of its release. Cytotoxic effects of zeolite-binase complexes toward colorectal cancer cells were compared with cytotoxicity of enzyme or zeolite. Our results contribute to the perspective development of binase-based complexes for therapy of colorectal cancer.

## Materials and Methods

### Binase

The guanyl-preferring RNase from *B. pumilus*, binase (monomer of 12.2 kDa, 109 amino acid residues, pI 9.5), was isolated from culture fluid of native binase producer as homogenous protein using the three-step procedure described earlier (Dudkina et al., 2016). The binase catalytic activity was determined by measurement of high-polymeric yeast RNA hydrolysis products according to modified method of Anfinsen (Kolpakov and Il’inskaia, 1999).

### Zeolites

Chabazite [(Ca,Na_2_,K_2_,Mg)Al_2_Si_4_O_12_ × 6H_2_O], the mineral of trigonal syngony, crystallizes in the triclinic crystal system with typically rhombohedral shaped crystals. The crystals are typically twinned, and both contact twinning and penetration twinning may be observed. Crystals of local chabazite up to 5 cm in size have pseudocubic forms, are pale orange with pearly tint and are characterized by a high degree of stoichiometry. In our study we used the samples from Sokolovo-Sarbaisky ore complex, Kazakhstan.

Clinoptilolite [(Na,K,Ca)_2-3_Al_3_(Al,Si)_2_Si_13_O_36_ × 12H_2_O] forms as white to reddish tabular monoclinic tectosilicate crystals. We used samples from Tatar-Shatrashan deposit of zeolite-bearing rocks, Russia. This mineral of the monoclinic syngony exists on the specified deposit in a fine-dispersed state, which is part of a polymineral aggregate consisting of a clayey and siliceous phase (the so-called zeolite-bearing rock). The maximum amount of zeolite in this unit can reach 50%.

Natrolite [Na_2_Al_2_Si_3_O_10_ × 2H_2_O] often occurs in compact fibrous aggregates, the fibers having a divergent or radial arrangement. Natrolite is a mineral of rhombic syngony, in the zones of metasomatic processing of alkaline igneous rocks of the Kola Peninsula forms large (up to 1 m) mono- and polycrystalline aggregates of snow-white color with a characteristic silky shine. We used natrolite from the Khibiny Mountains of the Kola Peninsula, Russia.

### Loading/Unloading Procedure

Zeolite powder obtained after grinding in an electric mill was treated with concentrated filtered hydrochloric acid HCl to remove various impurities, washed with MQ-water and dried using dry heat oven at 160°C. Each sample (5 mg) was mixed with binase solution in 96% ethanol (1 mg/ml), thoroughly vortexed (V-1 plus, Biosan, Latvia) until a homogeneous suspension, and then sonicated in ice for 5 min, 35 kHz, 130 W (Sapphire, Russia) to disintegrate the aggregates. Afterward samples were incubated for 2 h with gentle shaking (Mini Rocker-Shaker MR-1, Biosan, Latvia) at 25°C. To estimate the part of non-immobilized binase, protein concentration by optical density at 280 nm and RNase catalytic activity of the supernatant obtained after centrifugation (5 min, 4300 g, Eppendorf 5415R, Germany) were measured. The sediments were dried at 50°C and stored at room temperature. To analyze the release of immobilized enzyme, the samples were suspended in MQ-water and incubated at room temperature for 2, 4, or 6 h. After centrifugation, the binase catalytic activity and protein concentrations were measured in the supernatant.

### Cell Cultures

Colon adenocarcinoma cells (Caco2) were obtained from Russian cell culture collection (Saint-Petersburg, Russia). Cells were grown in RPMI 1640 medium supplemented with penicillin (100 U/mL), streptomycin (100 U/mL), 2 mM glutamine (Sigma-Aldridge, United States), and 10% fetal bovine serum (HyClone, United States) at 37°C in a humidified atmosphere with 5% CO_2_. Cells were seeded into 96-well plates and grown 12 h; then tested samples dissolved in fresh medium were added into plates. After 24 and 48 h of incubation the MTT assay was performed.

### MTT-Assay

Cell viability was measured according to mitochondrial dehydrogenase activity tested by standard procedure based on the reduction of MTT tetrazolium dye. Cells (10^4^ per well in 96-well plate, CELLTREAT Scientific Products, United States) have grown overnight, then cultural fluid was discarded and fresh medium with test samples (or with an equivalent volume of water for negative control) was added. After 24 and 48 h culture medium was replaced with dimethyl sulfoxide (Sigma-Aldrich, United States) to dissolve formazan crystals, when absorption was measured at 570 nm (xMark, Bio-Rad, United States). As a positive control inducing cell death 1% Triton was used.

### Transmission Electron Microscopy

Zeolite samples with 96% ethanol solution were sonicated during 10 min, 35 kHz, 130 W (Sapphire, Russia) to disintegrate the aggregates. A droplet of diluted zeolite samples was placed onto carbon-coated grids and left to evaporate. Specimens were inspected using a Hitachi HT7700 Exalens transmission electron microscope (Hitachi High-Tech Science Corporation, Japan) at resolution 1.4 Å. TEM bright field images were recorded at 100 kV accelerating voltage using a AMT XR-81 CCD camera (3296 × 2742, 8 megapixel, 5.5 mm pixel size).

### Statistics

Statistical data analysis and plotting were performed by means of GraphPad Prism6 software (United States). The statistically significant level was taken as *p* ≤ 0.05.

## Results

**FIGURE 1:**
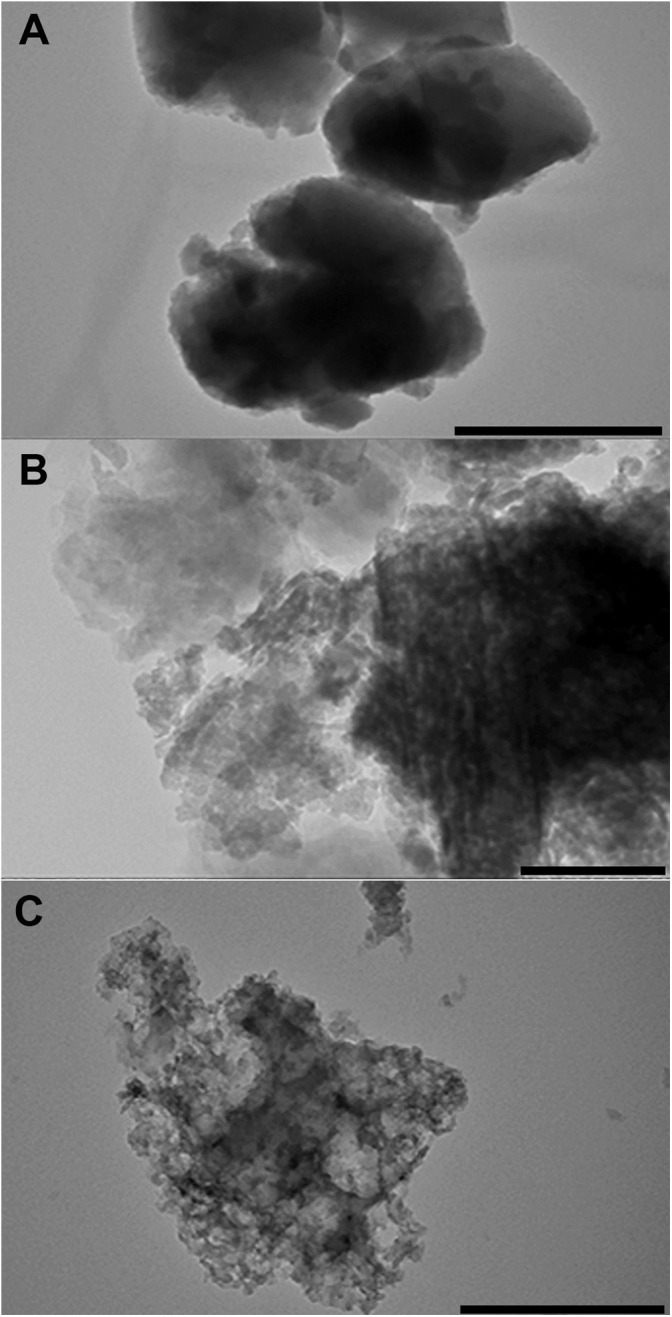
TEM images of three different zeolites (Chabazite – **A**, Clinoptilolite – **B**, and Natrolite – **C**) grinded in an electric mill up to particles of micrometer size. bar = 200 μm.

Chabazite is morphologically represented by small oval or pseudocubic particles with a diameter about 100∼200 nm aggregated into regular round-shaped particles (Ø ∼2 μm). The same small particles are typical for clinoptilolite, but they form amorphous structures of different size. Small particles of natrolite are partially leafed or polygonal forms (Figure 1).

Finely crushed samples of three different zeolites in the form of micrometer particles were used for binase immobilization. Initially, during the selection of binase loading conditions we used aqueous solution of enzyme but the measutment of unloaded protein concentration and RNase activity of the supernatant showed the lack of enzyme immobilization. Therefore we used 96% ethanol to solve the enzyme before loading. RNase activity in ethanol solution was almost the same as in water, 1.116 ± 0.013 × 10^6^ units/mg and 1.588 ± 0.020 × 10^6^ units/mg, correspondingly. More than 80% of the protein was found to adsorbe on all zeolites, whereby residual catalytic activity measured in supernatant was very low. The best results were obtained with chabazite (Table 1). Full release of binase from chabazite takes 6 h, for clinoptilolite this time period is 4 h. Natrolite kept residual amount of protein more than 6 h. The main part of protein (more that 80% of immobilized one) released from all three zeolites was found in solution already after 2h of incubation (Table 2). RNase activity of released binase was comparable to the activity of pure binase in water. Staying in natrolite reduced the catalytic activity of the enzyme released after 2 h up to 57%. This effect disappeared after 4 h of incubation. Opposite, staying inside chabazite slightly activated the binase catalytic activity (Table 2).

**Table 1:** The amount of binase loaded onto zeolite and silica samples from ethanol solution^a^.

Zeolite sample	Loaded enzyme, %	
	
	Measured by protein concentration	Measured by catalytic activity
Chabazite	86.9 ± 1.9	100 ± 0.8
Clinoptilolite	85.4 ± 0.8	99 ± 0.9
Natrolite	83.9 ± 1.2	98 ± 1.2


*^*a*^The protein concentration was measured using absorption spectrophotometry at 280 nm (SmartSpec Plus, Bio-Rad, United States); the catalytic activity was estimated as described in section “Materials and Methods.” The initial concentration of protein (1 mg/ml) and RNase catalytic activity in ethanol solution (1.116 ± 0.013 × 10^*6*^ units/mg) used for enzyme loading was taken for 100%. Values are means ± SD. Experiments were performed in triplicate with five independent replications in each series.*

**Table 2:** The amount of binase protein released from zeolite samples into MQ-water and catalytic activity of the released enzyme.

Zeolite sample	Released protein^a^/catalytic activity^b^, %		
	
Time of incubation, h	2	4	6
Chabazite	90.7 ± 0.7**/**147 ± 24	93.1 ± 1.0**/**133 ± 18	100 ± 0.4**/**n^c^
Clinoptilolite	98.0 ± 0.9**/**115 ± 17	100 ± 0.5**/**97 ± 19	100 ± 0.6**/**n
Natrolite	82.7 ± 0.5**/**57 ± 21	87.6 ± 0.3**/**112 ± 16	98.0 ± 0.4**/**n


*^*a*^The amount of loaded binase was taken for 100%. ^*b*^Catalytic activity RNase dissolved in MQ-water (1.588 ± 0.02 × 10^*6*^ units/mg) was taken for 100%. ^*c*^n – Not measured. Values are means ± SD. Experiments were performed in triplicate with five independent replications in each series.*

**FIGURE 2:**
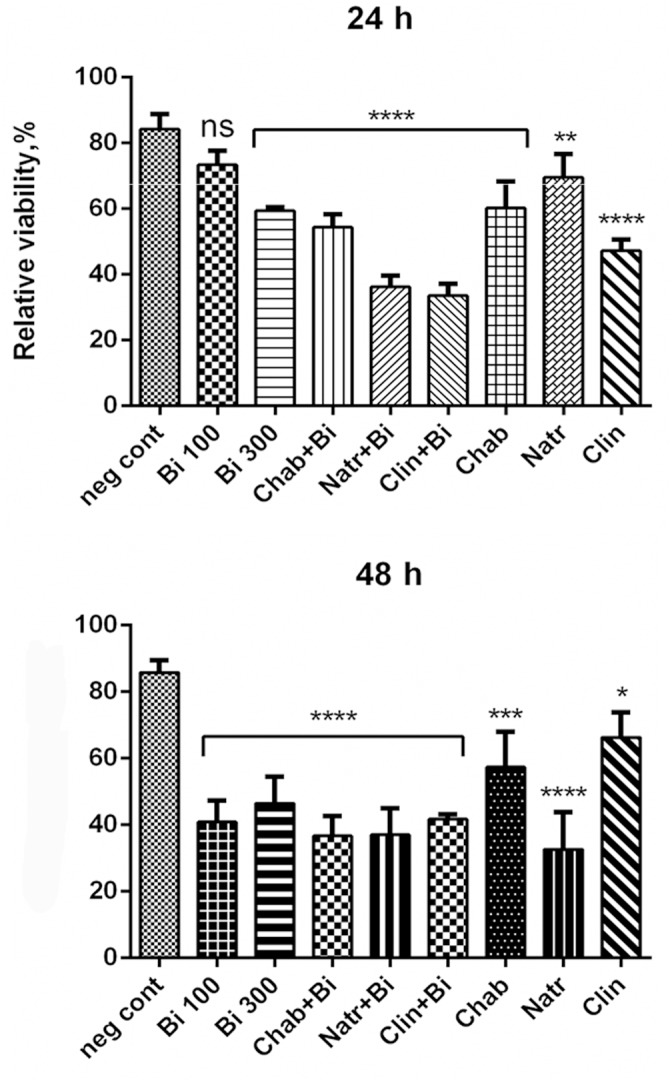
Cytotoxicity of binase-loaded zeolites (300 μg/ml), pure zeolites (300 μg/ml) and pure binase at two concentrations, 100 and 300 μg/ml, toward human colon adenocarcinoma Caco2 cells. Data represent mean ± SEM of three independent experiments; ^∗^*P* < 0.05, ^∗∗^*P* < 0.03, ^∗∗∗^*P* < 0.014, and ^∗∗∗∗^*P* < 0.01, correspondingly vs. negative control obtained by adding water volume equal to volume of zeolite suspension and binase solution; ns, non-significant. Cell viability without any additives was taken as 100%.

The cytotoxicity of pure zeolites and zeolites loaded with binase was studied on human colon adenocarcinoma cell line Caco2. Each type of zeolites (300 μg/ml) was examined on possible toxic effects after 24 and 48h of incubation with growing cells (Figure 2). After addition of the same amount of water used for zeolite suspension the cells viability reduced on 18% compared to growth without any supplements. Pure chabazite inhibited cell viability by less than 40% during all time of cultivation, clinoptilolite showed inhibitory effect of approximately 50% after 24 h decreased after 48 h up to 30%. Natrolite was more toxic, its inhibitory effect increased from 30% at 24 h to 70% at 48 h of cell growth.

Pure binase at concentration 100 μg/ml reduced cell viability by 60% only after 48 h of incubation. Higher concentration (300 μg/ml) affected cell viability already after 24 h (inhibition reached 40%), after 48 h inhibitory effect was 58% (Figure 2). Binase immobilized in natrolite or clinoptilolite increased their toxicity during 24 h, then this increase for natrolite, but not for clinoptilolite, was abolished. Complexes of clinoptilolite as well as chabazite with binase always demonstrated enhanced toxicity in comparison with binase and zeolites separately (Figure 2).

## Discussion

Binase possesses selective toxicity toward certain tumor cells *in vitro* (Mitkevich et al., 2011, 2013, 2015; Zelenikhin P. et al., 2016; Zelenikhin P.V. et al., 2016; Makeeva et al., 2017) and *in vivo* (Mironova et al., 2013; Sen’kova et al., 2014).

The expression of certain oncogenes (*ras, kit, AML1-ETO*) is a marker of tumor cells susceptibility to binase apoptogenic action. In some cases RNases catalytic activity may be an important factor for their cytotoxicity manifestation (Ilinskaya and Vamvakas, 1997; Makarov and Ilinskaya, 2003). However, the sensitivity of malignant breast cancer cells to binase apoptosis inducing effect was not shown to correlate with the level of cellular RNA catalytic degradation (Zelenikhin P. et al., 2016). This effect was also demonstrated for oncogene *kit* transformed cells (Mitkevich et al., 2010). Using quantitative RT-PCR with RNA samples isolated from the binase-treated transgenic myeloid progenitor cells FDC-P1-N822K expressing the activated *kit*-oncogene (mutation Asn822Lys), we have found that the amount of mRNA of the kit oncogene gene was reduced by half. This means that binase effect to tumor cells is specific and is determined by presence of cells specific molecular targets, which can be certain RNA as well as proteins, in particular, RAS (Ilinskaya et al., 2016).

Therefore, its delivery and prolonged action could have benefits during application against cancer.

Zeolites are a group of calcium and sodium aqueous aluminosilicates similar in composition and properties. In the gut, these silicates could act as adsorbents, catalysts, detergents or anti-diarrheic agents to their absorption potential and ion-exchanger properties. Zeolites themselves are widely used in agriculture as adsorbents. In animals, zeolite supplementation of feed resulted in a reduction in number of poultry pathogens without damaging the beneficial bacteria (Prasai et al., 2017). Dietary administration of small particle size clinoptilolite can effectively reduce concentration of aflatoxins in dairy cattle milk (Katsoulos et al., 2016). So, the detoxificant role of zeolites is already evident in agro and in zoothecnical fields [for review see Laurino and Palmieri, 2015). We started our study from two simple approvals. First, clinoptilolite application in medicine is allowed, preparations “Tribo Ming” (Croatia), and “Nov” (Russia) based on this zeolite are available for purchase in pharmacies. Natural clinoptilolite with enhanced physicochemical properties is the basis of the dietary supplements Megamin and Lycopenomin, which have demonstrated antioxidant activity in humans (Ivkovic et al., 2004).

We have also studied the possibility of other zeolites, chabazite and natrolite to serve as carriers for binase. Chabazite was studied previously as an agent for wastewater purification (Lee et al., 2016; Montégut et al., 2016), natrolite was described as an environmentally benign catalyst (Nasrollahzadeh et al., 2017). Our results have shown that all three zeolites used at this study have the possibility to absorb binase, an antitumor bacterial protein. The zeolites crystalline structure is formed by tetrahedral SiO_2/4_ and AlO_2/4_, groups, joined by common vertices into three-dimensional framework, penetrated by cavities and channels 2–15 Å in size. The surface of zeolites has a negative charge, compensated by counterions (metal cations, ammonium, and other ions) and water molecules. Washing the zeolites with acid allowed us to get rid of carbonate impurities, and the subsequent washing with water and alcohol removed counterions and released the negative charge necessary for sorption of cationic binase (PI 9.5) due to electrostatic interactions.

We found the conditions suitable for loading more than 80% of protein from ethanol solution during 2 h with gentle shaking by room temperature. Natrolite demonstrated slowly decreasing absorption ability compared to clinoptilolite and chabazite. It probably could be connected to its fibrous nature (Table 1). On the other hand, binase was released from natrolite more slowly than from clinoptilolite and chabazite and did not reached 100% output during 6 h (Table 2). It could be a positive fact for prolonged binase action, but natrolite itself possessed cytotoxicity increasing along the time of incubation with the cells up to high value about 40% with the same cytotoxicity value as pure binase. Therefore, clinoptilolite and chabazite have some preferences for use as binase carriers. Complex of clinoptilolite with binase induced the cell death comparable to pure binse after 48 h, but during the first 24 h of incubation the release of binase from clinoptilolite induced higher cytotoxicity as pure binase (Figure 2). This data are in accordance with previously obtained results about the capacity of clinoptilolite to be useful in medicine. Zeolite-containing mixture (Hydryeast) maintaining mucosal immune homeostasis and epithelial integrity, is known to have a suppressive effect on colitis (Lyu et al., 2017). In humans, zeolite supplementation exerted beneficial effects on intestinal wall integrity and accompanied by mild anti-inflammatory effects in aerobically trained subjects (Lamprecht et al., 2015). Treatment of cancer-bearing mice and dogs with micronized zeolite clinoptilolite led to improvement of the overall health status, prolongation of life span and decrease of tumor size in some cases. Combined treatment with doxorubicin and clinoptilolite resulted in strong reduction of the pulmonary metastasis count increasing anticancer effects of doxorubicin (Zarkovic et al., 2003). Clinoptiolite is also used in water filters, to soil improvement, wastewater treatment and remidiation, in veterinary medicine (in gastrointestinal tract treatment). Chabazit does not have such widespread use.

So, our results could rise especial interest concerning a binase with chabazite complex. First of all, this complex was always more cytotoxic toward Caco2 cells then chabazite or binase themselves. Then, chabazite has low cytotoxicity. Finally, binase release from chabazite is time-dependent (Figure 2). Moreover, the catalytic activity of binase was slightly stimulated during staying inside of chabazite (Table 2) possibly due to interaction with cations released from this carrier. It means that chabazite-binase complex could be a perspective anticancer agent.

Binase cytotoxicity has grown with concentration increasing during 24 h of incubation. At 48 h of incubation, the difference in cytotoxicity of 100 and 300 μg/ml binase was not significant (Figure 2). This could be probably caused by the fact that absorption of binase by cells occured rather quickly, especially in first hours, and reached a practical maximum at 6 h. At this time (6 h) we previously described a permeability peak for trypan blue-labeled albumin macromolecule across cell membrane of cancer lung epithelial cell monolayers treated with RNase (Cabrera-Fuentes et al., 2013). Probably, during prolonged incubation, binase adsorption slows down, which leads to cytotoxicity of the two used concentrations differences leveling at 48 h incubation. Over time, we observed increasing toxicity of natrolite, which formed the needle-shaped fibrous aggregates, and damaging cells. Therefore, natrolit cannot be recommended as a carrier of potential therapeutic proteins.

Now, application of zeolites as materials for various therapeutic substances delivery include antitumor ones is intensively studied. The composition synthesized from naturally occurring non-toxic zeolites had a 100% kill rate within 72 h against buccal mucosa and lung squamous epithelial cell cancers and was non-toxic to healthy human cells (Kaufman, 2001). Zeolite-based nanoparticles used in generating time-controlled release of 5-fluorouracil from zeolite preparations showed anti-cancer effect toward Caco-2 monolayers (Spanakis et al., 2014). Earlier, we have demonstrated that binase-halloysite complex doubled anticancer efficiency of binase due to its perfect absorption by cells and longer release reducing the viability of human colon adenocarcinoma cells Colo320 by 60% (Khodzhaeva et al., 2017). The same level of toxicity toward human adenocarcinoma Caco2 cells was obtained for chabazite-binase complex. At the first time, we have shown that not only clinoptiolite but also chabazite could be used as carriers for new antitumor agents inducing prolonged cytotoxicity toward cancer cells. Moreover, chabazite could help to counteract oxidative stress in apparently healthy subjects exposed to different oxidative stress risk factors affecting the levels of different antioxidant enzymes (gluthatione peroxidase, superoxide dismutase, and gluthatione reductase (Dogliotti et al., 2012). Our results indicates that (a) the toxicity of chabazite is insignificant in magnitude and does not increase with time; (b) its complex with binase exhibits cytotoxicity increasing with time due to release of binase from the complex; (c) the level of complex toxicity is slightly higher in comparison with pure binase. These facts could open the prospect of using chabazite as a carrier for potential therapeutics proteins.

## Author Contributions

VK, OL, and OI planned the experiments. VK and PZ performed the experiments. OL and OI analyzed the data. VK and OI wrote the manuscript.

## Conflict of Interest Statement

The authors declare that the research was conducted in the absence of any commercial or financial relationships that could be construed as a potential conflict of interest.

## Footnotes

1 https://www.mskcc.org/cancer-care/integrative-medicine/herbs/zeolite

## References

[B1] Cabrera-Fuentes H. A. Aslam M. Saffarzadeh M. Kolpakov A. Zelenikhin P. Preissner K. T. (2013). Internalization of Bacillus intermedius Ribonuclease (BINASE) induces human alveolar adenocarcinoma cell death. *Toxicon* 69 219–226. 10.1016/j.toxicon.2013.03.015 23567038

[B2] Cabrera-Fuentes H. A. Zelenikhin P. V. Kolpakov A. I. Preissner K. Ilinskaya O. N. (2012). Comparative toxicity of binase towards tumor and normal cell. *Toxicon* 60 104–105. 10.1016/j.toxicon.2012.04.019

[B3] de Assis L. V. Locatelli J. Isoldi M. C. (2014). The role of key genes and pathways involved in the tumorigenesis of malignant mesothelioma. *Biochim. Biophys. Acta* 1845 232–247. 10.1016/j.bbcan.2014.01.008 24491449

[B4] Dogliotti G. Malavazos A. E. Giacometti S. Solimene U. Fanelli M. Corsi M. M. (2012). Natural zeolites chabazite/phillipsite/analcime increase blood levels of antioxidant enzymes. *J. Clin. Biochem. Nutr.* 50 195–198. 10.3164/jcbn 22573920 PMC3334371

[B5] Dudkina E. Ulyanova V. Shah Mahmud R. Khodzhaeva V. Dao L. Vershinina V. (2016). Three-step procedure for preparation of pure Bacillus altitudinis ribonuclease. *FEBS Open Bio.* 6 24–32. 10.1002/2211-5463.12023 27047739 PMC4794795

[B6] Elmore A. R. (2003). Final report on the safety assessment of aluminum silicate, calcium silicate, magnesium aluminum silicate, magnesium silicate, magnesium trisilicate, sodium magnesium silicate, zirconium silicate, attapulgite, bentonite, Fuller’s earth, hectorite, kaolin, lithium magnesium silicate, lithium magnesium sodium silicate, montmorillonite, pyrophyllite, and zeolite. *Int. J. Toxicol.* 22(Suppl. 1), 37–102. 10.1080/10915810305085 12851164

[B7] Ilinskaya O. Decker K. Koschinski A. (2001). Bacillus intermedius ribonuclease as inhibitor of cell proliferation and membrane current. *Toxicology* 156 101–107. 10.1016/s0300-483x(00)00335-8 11164612

[B8] Ilinskaya O. Singh I. Dudkina E. Ulyanova V. Kayumov A. Barreto G. (2016). Direct inhibition of oncogenic KRAS by Bacillus pumilus ribonuclease (binase). *Biochim. Biophys. Acta* 1863 1559–1567. 10.1016/j.bbamcr.2016.04.005 27066977

[B9] Ilinskaya O. N. Dreyer F. Mitkevich V. A. Shaw K. L. Pace C. N. Makarov A. A. (2002). Changing the net charge from negative to positive makes ribonuclease Sa cytotoxic. *Protein Sci.* 10 2522–2525. 10.1110/ps.0216702 12237473 PMC2373699

[B10] Ilinskaya O. N. Vamvakas S. (1997). Nephrotoxic effects of bacterial ribonucleases in the isolated perfused rat kidney. *Toxicology* 120 55–63. 10.1016/s0300-483x(97)03639-1 9160109

[B11] Ivkovic S. Deutsch U. Silberbach A. Walraph E. Mannel M. (2004). Dietary supplementation with the tribomechanically activated zeolite clinoptilolite in immunodeficiency: effects on the immune system. *Adv. Ther.* 21 135–147. 10.1007/bf02850340 15310086

[B12] Katsoulos P. D. Karatzia M. A. Boscos C. Wolf P. Karatzias H. (2016). In-field evaluation of clinoptilolite feeding efficacy on the reduction of milk aflatoxin M1 concentration in dairy cattle. *J. Anim. Sci. Technol.* 58 24. 10.1186/s40781-016-0106-4 27413536 PMC4943012

[B13] Kaufman H. (2001). *Epithelial Cell Cancer Drug.* U.S. Patent No 6288045. Stow, OH: Lifelink Pharmaceuticals Inc.

[B14] Khodzhaeva V. Makeeva A. Ulyanova V. Zelenikhin P. Evtugyn V. Hardt M. (2017). Binase immobilized on halloysite nanotubes exerts enhanced cytotoxicity toward human colon adenocarcinoma cells. *Front. Pharmacol.* 8:631. 10.3389/fphar.2017.00631 28955235 PMC5600959

[B15] Kolpakov A. I. Il’inskaia O. N. (1999). The optimization of a method for determining RNAse activity by using high-polymer RNA. *Klin. Lab. Diagn.* 5 14–16. 10399432

[B16] Lamprecht M. Bogner S. Steinbauer K. Schuetz B. Greilberger J. F. Leber B. (2015). Effects of zeolite supplementation on parameters of intestinal barrier integrity, inflammation, redoxbiology and performance in aerobically trained subjects. *J. Int. Soc. Sports Nutr.* 12:40. 10.1186/s12970-015-0101-z 26500463 PMC4617723

[B17] Laurino C. Palmieri B. (2015). Zeolite: “the magic stone”; main nutritional, environmental, experimental and clinical fields of application. *B. Nutr. Hosp.* 32 573–581. 10.3305/nh.2015.32.2.8914 26268084

[B18] Lee K. Y. Kim K. W. Park M. Kim J. Oh M. Lee E. H. (2016). Novel application of nanozeolite for radioactive cesium removal from high-salt wastewater. *Water Res.* 95 134–141. 10.1016/j.watres.2016.02.052 26990838

[B19] Lyu W. Jia H. Deng C. Saito K. Yamada S. Kato H. (2017). Zeolite-containing mixture supplementation ameliorated dextran sodium sulfate-induced colitis in mice by suppressing the inflammatory bowel disease pathway and improving apoptosis in colon mucosa. *Nutrients* 9:E467. 10.3390/nu9050467 28481231 PMC5452197

[B20] Makarov A. A. Ilinskaya O. N. (2003). Cytotoxic ribonucleases: molecular weapons and their targets. *FEBS Lett.* 540 15–20. 10.1016/s0014-5793(03)00225-4 12681476

[B21] Makeeva A. Rodriguez-Montesinos J. Zelenikhin P. Nesmelov A. Preissner K. T. Cabrera-Fuentes H. A. (2017). Antitumor macrophage response to bacillus pumilus ribonuclease (Binase). *Mediators Inflamm.* 2017:4029641. 10.1155/2017/4029641 28804220 PMC5540387

[B22] Mironova N. L. Petrushanko I. Y. Patutina O. A. Sen’kova A. V. Simonenko O. V. Mitkevich V. A. (2013). Ribonuclease binase inhibits primary tumor growth and metastases via apoptosis induction in tumor cells. *Cell Cycle* 12 2120–2131. 10.4161/cc.25164 23759588 PMC3737314

[B23] Mitchell S. Pinar A. B. Kenvin J. Crivelly P. Kärger J. Pérez-Ramírez J. (2015). Structural analysis of hierarchically organized zeolites. *Nat. Commun.* 6:8633. 10.1038/ncomms9633 26482337 PMC4667694

[B24] Mitkevich V. A. Ilinskaya O. N. Makarov A. A. (2015). Antitumor RNases: killer’s secrets. *Cell Cycle* 14 931–932. 10.1080/15384101.2015.1010972 25714843 PMC4614991

[B25] Mitkevich V. A. Kretova O. V. Petrushanko I. Y. Burnysheva K. M. Sosin D. V. Simonenko O. V. (2013). Ribonuclease binase apoptotic signature in leukemic Kasumi-1 cells. *Biochimie* 95 1344–1349. 10.1016/j.biochi.2013.02.016 23499289

[B26] Mitkevich V. A. Petrushanko I. Y. Kretova O. V. Zelenikhin P. V. Prassolov V. S. Tchurikov N. A. (2010). Oncogenic c-kit transcript is a target for binase. *Cell Cycle* 9 2674–2678. 10.4161/cc.9.13.12150 20581458

[B27] Mitkevich V. A. Petrushanko I. Y. Spirin P. V. Fedorova T. V. Kretova O. V. Tchurikov N. A. (2011). Sensitivity of acute myeloid leukemia Kasumi-1 cells to binase toxic action depends on the expression of KIT and ÀML1-ETO oncogenes. *Cell Cycle* 10 4090–4097. 10.4161/cc.10.23.18210 22101339

[B28] Montégut G. Michelin L. Brendlé J. Lebeau B. Patarin J. (2016). Ammonium and potassium removal from swine liquid manure using clinoptilolite, chabazite and faujasite zeolites. *J. Environ. Manag.* 167 147–155. 10.1016/j.jenvman.2015.11.027 26686066

[B29] Nasrollahzadeh M. Sajadi S. M. Maham M. Dasmeh H. R. (2017). In situ green synthesis of Cu nanoparticles supported on natural Natrolite zeolite for the reduction of 4-nitrophenol, congo red and methylene blue. *IET Nanobiotechnol.* 11 538–545. 10.1049/iet-nbt.2016.0143 28745286 PMC8676655

[B30] Prasai T. P. Walsh K. B. Bhattarai S. P. Midmore D. J. Van T. T. Moore R. J. (2017). Zeolite food supplementation reduces abundance of enterobacteria. *Microbiol. Res.* 195 24–30. 10.1016/j.micres.2016.11.006 28024523

[B31] Sen’kova A. V. Mironova N. L. Patutina O. A. Mitkevich V. A. Markov O. V. Petrushanko I. Y. (2014). Ribonuclease binase decreases destructive changes of the liver and restores its regeneration potential in mouse lung carcinoma model. *Biochimie* 101 256–259. 10.1016/j.biochi.2014.02.006 24565811

[B32] Spanakis M. Bouropoulos N. Theodoropoulos D. Sygellou L. Ewart S. Moschovi A. M. (2014). Controlled release of 5-fluorouracil from microporous zeolites. *Nanomedicine* 10 197–205. 10.1016/j.nano.2013.06.016 23916887

[B33] Suri S. Panda B. Javed S. Mohd A. (2007). RNase: a novel enzyme for treatment of cancers. *Internet J. Oncol.* 5 1–5.

[B34] Zarkovic N. Zarkovic K. Kralj M. Borovic S. Sabolovic S. Blazi M. P. (2003). Anticancer and antioxidative effects of micronized zeolite clinoptilolite. *Anticancer. Res.* 23 1589–1595. 12820427

[B35] Zelenikhin P. Pukhovskaya V. Garipov A. Makeeva A. Sokolova E. Ilinskaya O. (2016). Obvious and hidden reasons of breast cancer cell sensitivity to antitumor RNase. *BioNanoScience* 6 528–533. 10.1007/s12668-016-0269-y

[B36] Zelenikhin P. V. Makeeva A. V. Nguen T. N. Siraj Y. A. Ilinskaya O. N. (2016). The combined action of binase and bleomycin on human lung adenocarcinoma cells. *Biochem. Suppl. Ser. B Biomed. Chem.* 10 87–90. 10.1134/S1990750816010121 27420619

